# Therapeutic Drug Monitoring of Antifungal Drugs: Another Tool to Improve Patient Outcome?

**DOI:** 10.1007/s40121-020-00280-y

**Published:** 2020-02-05

**Authors:** Antonio Vena, Patricia Muñoz, Miriam Mateos, Jesus Guinea, Alicia Galar, Federico Pea, Ana Alvarez-Uria, Pilar Escribano, Emilio Bouza

**Affiliations:** 1 Clinical Microbiology and Infectious Diseases, Hospital General Universitario Gregorio Marañón, Madrid, Spain; 2 Instituto de Investigación Sanitaria Hospital Gregorio Marañón, Madrid, Spain; 3 Department of Health Sciences, Infectious Disease Clinic, University of Genoa and Hospital Policlinico San Martino-IRCCS, Genoa, Italy; 4 Medicine Department, School of Medicine, Universidad Complutense de Madrid, Madrid, Spain; 5 CIBER Enfermedades Respiratorias- CIBERES (CB06/06/0058), Madrid, Spain; 6 Institute of Clinical Pharmacology, Santa Maria della Misericordia University Hospital of Udine, ASUIUD, Udine, Italy

**Keywords:** Antifungals, Azoles, Clinical outcome, Echinocandins, Invasive fungal infections

## Abstract

**Introduction:**

This study aimed to examine the relationship among adequate dose, serum concentration and clinical outcome in a non-selected group of hospitalized patients receiving antifungals.

**Methods:**

Prospective cross-sectional study performed between March 2015 and June 2015. Dosage of antifungals was considered adequate according to the IDSA guidelines, whereas trough serum concentrations (determined with HPLC) were considered adequate as follows: fluconazole > 11 µg/ml, echinocandins > 1 µg/ml, voriconazole 1–5.5 µg/ml and posaconazole > 0.7 µg/ml.

**Results:**

During the study period, 84 patients (65.4% male, 59.6 years) received antifungals for prophylaxis (40.4%), targeted (31.0%) and empirical therapy (28.6%). The most frequent drug was micafungin (28/84; 33.3%) followed by fluconazole (23/84; 27.4%), voriconazole (15/84; 17.9%), anidulafungin (8/84; 9.5%), posaconazole (7/84; 8.3%) and caspofungin (3/84; 3.6%). Considerable interindividual variability was observed for all antifungals with a large proportion of the patients (64.3%) not attaining adequate trough serum concentrations, despite receiving an adequate antifungal dose. Attaining the on-target serum antifungal level was significantly associated with a favorable clinical outcome (OR = 0.02; 95% CI 0.01–0.64; *p* = 0.03), whereas the administration of an adequate antifungal dosage was not.

**Conclusions:**

With the standard antifungal dosage, a considerable proportion of patients have low drug concentrations, which are associated with poor clinical outcome.

## Key Summary Points

**Table:** 

Adequate treatment of invasive fungal infections (IFIs) requires proper drug selection and proper dosing of antifungal drugs.
Triazoles or echinocandins are the most commonly used drugs for preventing or treating IFI, and systematic therapeutic drug monitoring (TDM) of antifungals is not considered routinely necessary.
We aimed to examine the relationship among adequate dose, serum concentration and clinical outcome in a non-selected group of hospitalized patients receiving antifungals.
We found that with a standard antifungal dosage, a considerable proportion of patients have low drug concentrations, which was associated with poor clinical outcome.

## Introduction

Invasive fungal infections (IFIs) remain a major clinical concern because of their increasing incidence, high morbidity and mortality rates [1, 2]. Adequate treatment requires proper drug selection and proper dosing of antifungal drugs [3–5].

Triazoles and echinocandins are the most commonly used drugs for preventing or treating IFI [6], and failure to achieve adequate serum concentrations has been advocated as a possible cause of poor outcomes [6, 7], emergence of resistance [8] and toxicity [9, 10]. Nevertheless, systematic therapeutic drug monitoring (TDM) of antifungals is not considered routinely necessary [6], mainly because of the belief that an adequate antifungal serum concentration is usually achieved by prescribing fixed doses according to international guidelines [11]. Unfortunately, evidence supporting this assumption is scarce and mainly related to specific groups of patients [12–15] or antifungal class [16–26].

The aim of this study was to determine whether doses of antifungal drugs accurately predict an adequate serum concentration in a non-selected group of hospitalized patients. We also tried to evaluate the impact of inadequate dosage or inadequate antifungal serum concentrations on clinical outcome.

## Materials and Methods

### Study Design and Patient Inclusion Criteria

This was a prospective observational cross-sectional study performed from March 2015 to June 2015 in our 1550-bed tertiary care hospital that serves a population of approximately 715,000 inhabitants. It is a referral center for solid organ transplantation, heart surgery, stem cell transplantation and HIV/AIDS care.

During the study period, the list of the patients who were starting systemic antifungal treatment was received daily from the pharmacy department. The choice of the antifungal agent as well as dosing was made by the attending physicians who were not aware of the study design. No feedback was maintained with them until the end of the study.

All consecutive non-selected adult patients who received a systemic triazole or an echinocandin for prophylaxis or treatment (either empirical or targeted) of IFI were included in the study if they gave their written informed consent and a blood sample for TDM was drawn. According to the study protocol, each patient had one blood sample drawn at least 3 days post-initiation of treatment. Trough levels were obtained within 30 min before dosing. Adequacy of antifungal dosing and serum concentration as well as clinical outcome was evaluated at discharge of the patient.

The study was approved by the institutional review board of the Hospital General Universitario Gregorio Maranon (MICRO.HGUGM.2015-066) and was in accordance with the Declaration of Helsinki. Written informed consent was obtained from each participating patient.

### Data Collection and Definition

The following data were prospectively collected using a standardized case report form: sex; age; weight and height; Charlson comorbidity index; renal and hepatic function (serum creatinine and creatinine clearance); presence of extracorporeal devices such as continuous renal replacement therapy (CRRT); risk factors for IFI (i.e., presence of a central venous catheter, parenteral nutrition, corticosteroid therapy, recent surgery); indication for antifungal treatment; type of antifungal drug; dosage; microbiologic findings and clinical evolution of the patients.

IFI-related mortality was defined as a death that could be attributed to IFI as either the immediate or underlying cause.

### Clinical Outcome

Clinical outcome was considered favorable when the following criteria were fulfilled: completion of treatment course without broadening the antifungal spectrum or addition of another antifungal drug, no evidence of breakthrough IFI and/or no evidence of IFI-related mortality.

### Antifungal Drug Administration and Sample Collection

The adequacy of the antifungal dosage was defined according to current IDSA guidelines [27]. Dose adjustments for hepatic and/or renal dysfunction and drug-drug interactions were also considered when necessary.

Serum antifungal concentrations were determined with high-performance liquid chromatography. Samples were processed as previously described by Arendrup et al. for anidulafungin and caspofungin [28], Gordien et al. for triazoles [29] and Martens-Lobenhoffer et al. for micafungin [30]. According to pharmacokinetic data, the following trough serum concentrations were considered within therapeutic range: fluconazole > 11 µg/ml [15], echinocandins > 1 µg/ml [31], voriconazole 1–5.5 µg/ml [32] and posaconazole > 0.7 µg/ml [6, 33]. Precision and accuracy were assessed by performing replicate analysis of quality control samples against calibration standards.

### Statistical Analysis

The total number (and percentage) of cases with trough concentrations out of the therapeutic target for each antifungal drug was assessed. Continuous variables are presented as mean value (± SD) or median values (range) for normally or non-normally distributed data. Categorical variables are expressed as frequency and percentage.

To identify independent predictors of the trough serum antifungal concentration, we performed uni- and multivariate linear regression analyses, including all the variables significant at *p* ≤ 0.20 in the univariate analysis in the multivariate stepwise backward analysis. A multivariate logistic regression model was used to assess the independent effect of either the adequate serum antifungal concentration or the adequate antifungal dose according to guidelines on the outcome of patients with IFI. A forward stepwise approach was followed, including all those that were significant in the univariate analysis as candidate variables. The results are presented as adjusted odds ratios with 95% confidence intervals. All statistical procedures were performed using SPSS version 15.0 (SPSS Inc., Chicago, IL, USA).

## Results

Overall, 84 patients were included in the study. Most of them were male (*n* = 55, 65.4%), and the mean age (± SD) was 59.6 years (± 14.1). Hospital admission wards, main underlying diseases, associated risk factors and indications for antifungal therapy are summarized in Table 1.

**Table 1:** Demographic and clinical characteristics of patients

Characteristics	*N* = 84 (%)
Age, years, mean ± SD	59.6 ± 14.1
Male sex	55 (65.4)
Charlson comorbidity index, mean ± SD	4.0 ± 2.9
Hospital department	
Onco-hematology	28 (33.4)
Intensive care unit	22 (26.2)
Internal medicine	20 (23.7)
Surgical	14 (16.7)
IFI risk factors	
Leukemia/lymphoma	22 (26.2)
Solid organ cancer receiving chemotherapy/radiotherapy	15 (17.9)
Hematopoietic stem cell transplant	11 (13.1)
HIV infection	11 (13.1)
Central venous catheter	62 (73.8)
Surgery in the last 3 months	37 (44.0)
Corticosteroids in the previous 1 month	33 (39.3)
Total parenteral nutrition	28 (33.3)
Continuous renal replacement therapy	3 (6.4)
Indication for antifungal therapy	
Prophylaxis	34 (40.4)
Empirical therapy	24 (28.6)
Targeted therapy	26 (31.0)
Adequate AF dosage according to guidelines	76 (90.5)
Adequate AF serum concentration	54 (64.3)

*AF* antifungals, *BSI* bloodstream infection

Antifungal drugs were prescribed as prophylaxis in 34 patients (40.4%), targeted therapy in 26 (31.0%) and empirical therapy in 24 (28.6%). *Candida* bloodstream infection (*n* = 11), followed by pulmonary aspergillosis (*n* = 8) and intra-abdominal candidiasis (*n* = 7), was the most common proven IFI.

### Correlation of Appropriate Trough Serum Concentrations and Antifungal Dosage

Among the 84 patients, the most frequently used antifungal agent was fluconazole 400 mg (16/84, 19.0%) followed by voriconazole, (15/84, 17.8%), micafungin 100 mg (14/84, 16.7%), micafungin 50 mg (14/84, 16.7%), anidulafungin (8/84, 9.5%), posaconazole (7/84, 8.4%), fluconazole 200 mg (6/84, 7.1%) and caspofungin (3/84, 3.6%). According to current guidelines, antifungal dosages were classified as appropriate in 76 out of 84 patients (90.5%).

As shown in Table 2, we observed a large inter-individual variability in trough serum concentration with all drugs: echinocandins ranged from 0 to 7.2 µg/ml, fluconazole from 1.9 to 47.7 µg/ml, voriconazole from 1.1 to 11.0 µg/ml and posaconazole from 0.2 to 2.2 µg/ml. Therefore, an adequate exposure according to serum concentration was reached in only 54/84 cases (64.3%). The proportions of samples with on-target serum levels were as follows: anidulafungin (8/8; 100%), voriconazole (13/15, 86.7%), fluconazole 400 mg (12/17, 70.5%), caspofungin (2/3, 66.7%), micafungin 100 mg (9/14, 64.2%), posaconazole (4/7, 57.1%), micafungin 50 mg (5/14, 35.7%) or fluconazole 200 mg (1/6, 16.7%).

**Table 2:** Clinical data and adequacy of antifungal dose according to serum concentration and current guidelines

	Overall	Fluconazole 200 mg (*n* = 6)	Fluconazole 400 mg (*n* = 17)	Voriconazole	Posaconazole	Micafungin 50 mg (*n* = 14)	Micafungin	Anidulafungin	Caspofungin
	84			(*n* = 15)	(*n* = 7)		100 mg (*n* = 14)	100 mg (*n* = 8)	50 mg (*n* = 3)
Factors potentially influencing antifungal serum concentration									
Male sex	55 (65.4)	3 (50.0)	9 (52.9)	11 (73.3)	3 (42.9)	9 (64.3)	12 (85.7)	6 (75.0)	2 (66.7)
Weight (kg), median (range)	66 (40–117)	59 (40–68)	62 (40–88)	64 (40–96)	69 (56–78)	84.5 (48–87)	67 (44–95)	75 (71–79)	70 (68–117)
Body surface area	1.70 ± 0.34	1.60 ± 0.15	1.59 ± 0.46	1.63 ± 0.50	1.74 ± 0.13	1.73 ± 0.20	1.78 ± 0.25	1.86 ± 0.11	2.26 ± 0.23
CL_CR_ (ml/min), median (IQR)	89.0 (48.3–116.4)	87.2 (64.4–140.5)	84.1 (44.2–84.1)	82.1 (67.8–115.2)	86.5 (49.6–105.1)	109.0 (94.5–126.7)	47.2 (24.5–106.8)	72.9 (35.1–183.6)	103.3 (NE)
Serum albumin (mg/dl), median (IQR)	3.0 (2.5–3.5)	2.3 (2.0–2.5)	2.6 (2.0–3.0)	3.6 (3.3–4.0)	3.3 (2.8–3.6)	3.3 (3.0–3.9)	2.7 (2.4–3.2)	2.4 (2–2.9)	2.8 (NE)
Creatinine (mg/dl), median (IQR)	0.8 (0.6–1.1)	0.6 (0.4–0.8)	0.7 (0.5–0.9)	0.9 (0.6–0.94)	0.8 (0.7–1.4)	0.7 (0.5–0.9)	1.3 (0.8–2.5)	0.8 (0.6–2.5)	0.7 (NE)
Intensive care unit stay	22 (26.2)	1 (16.7)	3 (17.6)	1 (6.7)	1 (14.3)	0 (0)	7 (50.0)	6 (75.0)	3 (100)
Adequancy of serum antifungal concentration									
According to serum concentration	54 (64.3)	1 (16.7)	12 (70.6)	13 (86.7)	4 (57.1)	5 (35.7)	9 (64.3)	8 (100)	2 (66.7)
According to current guidelines	76 (90.5)	2 (33.3)	16 (94.1)	13 (86.7)	7 (100)	14 (100)	13 (92.9)	8 (100)	3 (100)
Serum AF concentration (µg/ml), median (range**)**	–	7.0 (1.9–15.3)	15.09 (4.3–47.0)	3.2 (1.1–11.0)	0.8 (0.2–2.2)	0.83 (0–2.27)	1.2 (0.6–7.13)	2.3 (1.1–2.8)	3.25 (0–4.7)

*AF* antifungals,* CL*_*cr*_ clearance creatinine*, IQR* interquartile range, *NE* not evaluable

When we specifically analyzed patients receiving an adequate dose of antifungal according to the current guidelines (76/84, 90.5%), we found that again only 67.1% of them (51/76) attained an adequate serum concentration. Of the remaining 8/84 (9.5%) patients (all receiving lower than recommended antifungal dosage), 3 attained the on-target serum antifungal level and five did not.

### Variables Associated with the Trough Level of Antifungals

Variables associated with the trough level of antifungals are shown in Table 3. Univariate analysis showed that age, male sex, weight, body surface and daily dose of antifungals were all variables associated with trough concentration by either augmenting the drug exposure (older age, daily dose) or lowering it (male sex, weight and body surface area). Multivariate analysis confirmed age and daily dose as factors associated with an increased drug exposure, whereas the body surface area correlated with a decrease in serum antifungal concentrations. We were not able to demonstrate a significant correlation between renal function and trough serum antifungal concentration.

**Table 3:** Uni- and multivariate analysis of variables associated with trough level of all antifungals (*n* = 84)

	Univariate analysis		Multivariate analysis	
	Unstandardized *β*- coefficient (95% CI)	*p*	Unstandardized *β*- coefficient (95% CI)	*p*
Age (years)	**0.20 (0.07, 0.33)**	**< 0.01**	**0.16 (0.06–027)**	**0.003**
Male sex	**− 4.29 (− 0.32, − 8.26)**	**0.03**	–	–
Weight (kg)	**-0.11 (− 0.21, − 0.01)**	**0.03**	–	–
Body surface area	**-6.31 (− 11.89, − 7.32)**	**0.03**	**− 10.25 (− 17.42, − 3.09)**	**0.006**
CL_CR_ (ml/min)	-0.02 (-0.04, 0.01)	0.19	–	–
Daily dose (mg/kg)	**0.04 (0.02–0.04)**	**< 0.01**	**0.03 (0.02–0.04)**	**0.001**
Intensive care unit stay	**− **2.41 (**− **6.79, 1.96)	0.27	–	–
Serum albumin, mg/dl	**− **1.62 (**− **4.4, 1.17)	0.25	–	–
Charlson comorbidity index	0.04 (0.31, 1.10)	0.26	–	–

*P* values < 0.05 are shown in bold

When we separately analyzed each antifungal class administered in our study, no associations were found between any variables and trough serum concentration for voriconazole, posaconazole, caspofungin and anidulafungin. However, the serum albumin level was correlated with the fluconazole serum concentration [unstandardized β-coefficient (*β*) (95% interval confidence), *β* = + 11.45 (1.95, 20.95), *p* = 0.02], whereas age, [*β* = + 0.16 (0.06, 0.27), *p* = 0.003], male sex [*β* = − 1.48 (0.57, 2.44), *p* < 0.01], daily dose [*β* = 0.03 (0.01, 0.47), *p* < 0.01] and serum albumin [*β* = + 0.72 (0.33, 1.48), *p* = 0.04] were the variables showing significant correlation with the trough micafungin concentration.

### Clinical Evolution

Overall, a favorable clinical outcome was observed in 77/84 patients (91.6%) following a median therapy duration of 15 days. Factors associated with a poor outcome in the univariate analysis were high Charlson comorbidity index (*p* = 0.016), previous treatment with corticosteroid therapy (*p* = 0.01) and total parenteral nutrition (*p* = 0.04). Adequate serum antifungal concentration tended to be associated with a favorable outcome (*p* = 0.09) (Table 4), whereas no correlation between clinical outcome and adequate antifungal dosage was observed in univariate analysis.

**Table 4:** Univariate and multivariate analysis for risk factors for poor clinical outcome

Characteristics	Good clinical outcome	Poor clinical outcome	*p*	Multivariate analysis	*p*
	(*n* = 77, %)	(*n* = 7, %)		OR (95% CI)	
Age, years, mean ± SD	59.8 ± 14.1	57.9 ± 15.7	0.73	–	–
Male sex, %	50 (64.9)	5 (71.4)	1	–	–
Charlson comorbidity index	**3.7 ± 2.5**	**6.3 ± 3.4**	**0.016**	0.6 (0.3–1.1)	0.09
IFI risk factors					
Leukemia/lymphoma	22 (28.6)	0	0.18	–	–
Solid organ cancer with chemotherapy/radiotherapy	13 (16.9)	2 (28.6)	0.6	–	–
Hematopoietic stem cell transplant	10 (13.0)	1 (14.3)	1	–	–
HIV infection	10 (13.0)	1 (14.3)	1	–	–
Central venous catheter	56 (72.7)	6 (85.7)	0.67		–
Surgery in the last 3 months	33 (42.9)	4 (57.1)	0.69	–	–
Corticosteroids within the previous month	**27 (35.1)**	**6 (85.7)**	**0.01**	14.6 (0.98–220.6)	0.06
Total parenteral nutrition	**23 (29.9)**	**5 (71.4)**	**0.04**	2.74 (0.28–26.39)	0.38
Continuous renal replacement therapy	3 (7.1)	0	1	–	–
Indication for antifungal therapy, %					
Prophylaxis	32 (41.6)	2 (28.6)	0.6	**–**	**–**
Empirical therapy	23 (29.9)	1 (14.3)	0.7	**–**	**–**
Targeted therapy	22 (28.6)	4 (57.1)	1	**–**	**–**
Adequate AF dosage according to guidelines, %	69 (89.6)	7 (100)	1	1.12 (0.03–40.6)	0.93
Adequate AF serum concentration, %	52 (67.5)	2 (28.6)	0.09	**0.02 (0.01–0.64)**	**0.03**
Final diagnosis (patients with targeted indication), %					–
*Candida* BSI	9 (11.7)	2 (28.6)	0.22	–	–
Invasive aspergillosis	6 (7.8)	2 (28.6)	0.13	–	–
Intra-abdominal candidiasis	7 (9.1)	0	1	–	–
Creatinine clearance	97.5 ± 73.2	82.9 ± 56.7	0.6	–	–
Length of antifungal therapy	33.2 ± 32.2	18.2 ± 20.1	0.31	–	–

*P* values < 0.05 are shown in bold

Multivariate analysis (Table 4) showed that attaining the on-target serum antifungal level was significantly associated with a favorable clinical outcome (OR = 0.02; 95% CI 0.01–0.64; *p* = 0.03). Conversely, the administration of an adequate antifungal dosage according to current guidelines was not associated with a favorable clinical outcome (OR = 1.12; 95% CI 0.03–40.6, *p* = 0.93).

**Fig. 1:**
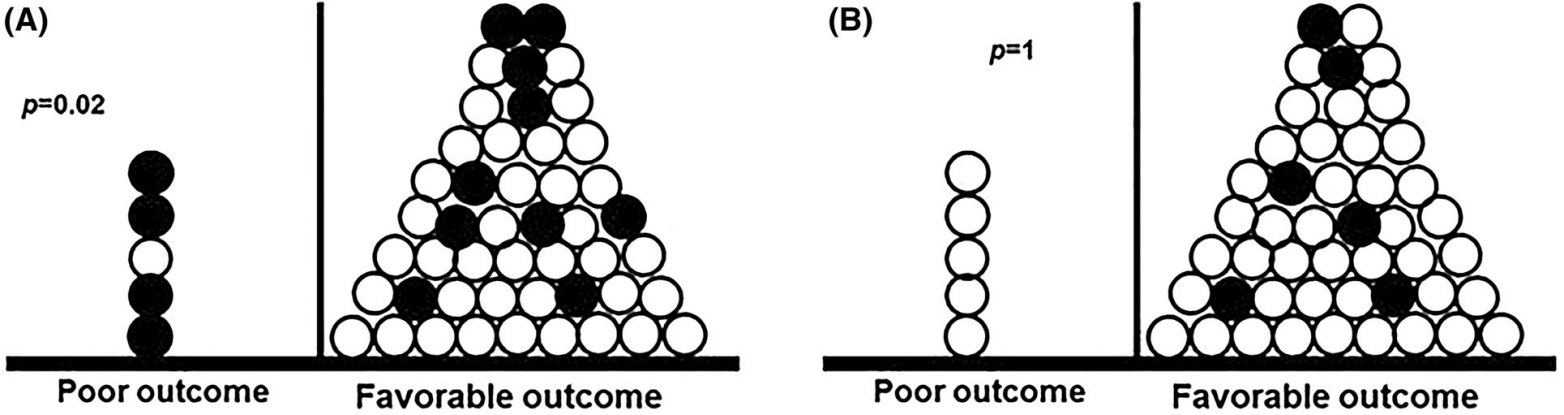
Comparison of poor or favorable clinical outcome according to adequacy of serum concentration (**a**) or dosage as suggested by current guidelines (**b**). Only patients receiving empirical or targeted therapy are included in this figure. Each patient is represented with a dot. Patients with inadequate antifungal exposure are shown with black dots, those with adequate AF exposure with white dots

**Fig. 2:**
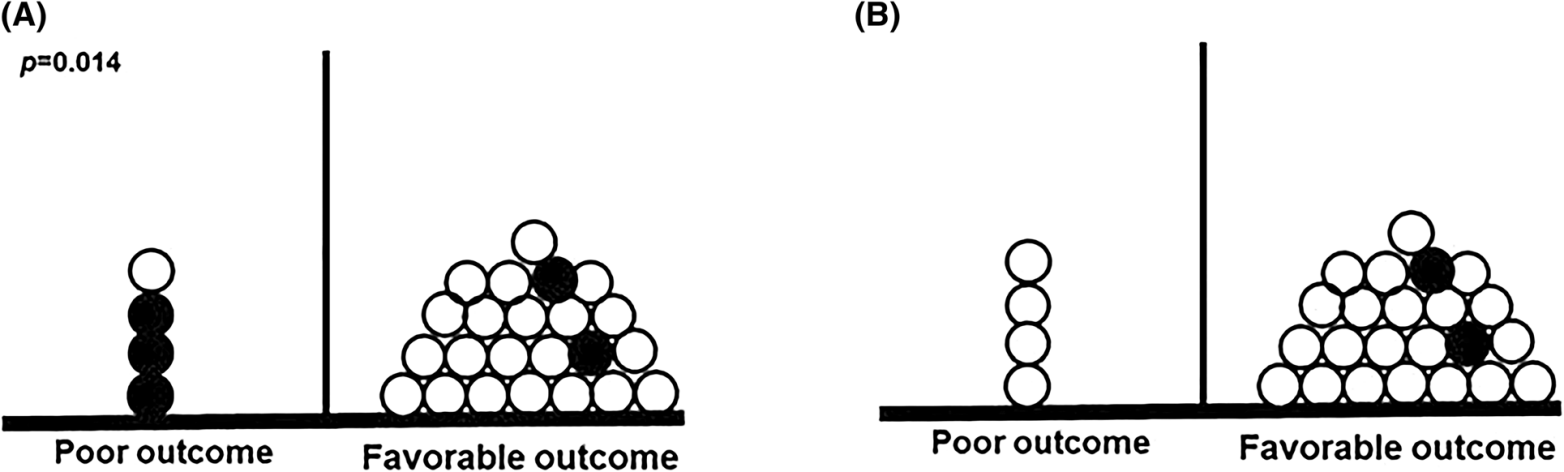
Comparison of poor or favorable clinical outcome according to adequacy of serum concentration (**a**) or dosage as suggested by current guidelines (**b**). Only patients receiving targeted therapy are included in this figure. Each patient is represented with a dot. Patients with inadequate antifungal exposure are shown with black dots, those with adequate antifungal exposure with white dots

When we performed an additional analysis including only patients with empirical or targeted therapy (Figs. 1 and 2), both uni- and multivariate analysis showed a strong correlation between favorable clinical outcome and serum antifungal drug concentration.

## Discussion

Our findings suggest that, in a non-selected group of hospitalized patients receiving triazoles or echinocandins, there is a poor correlation between guideline-based antifungal dosage and adequate serum drug concentrations, with many patients being outside the therapeutic target. Moreover, an adequate antifungal serum concentration seems to better predict the clinical outcome of the patients, thus advising performance of TDM of all antifungals at least in patients with hypoalbuminemia.

The mortality of patients with IFI is reported to be between 20 and 50% [6]. Risk factors for poor prognosis include the specific type of invasive fungal infection [34] and patient- or treatment-related factors (older age, neutropenia, malignancies, liver disease, delay in appropriate treatment) [34, 35]. Until recently, the effect of therapeutic drug monitoring was not perceived as a need for improving prognosis in patients receiving adequate antifungal doses [27].

Both the IDSA [27] and the British Society of Medical Mycology guidelines [33] recommend systematic TDM in patients receiving posaconazole or voriconazole because of their pharmacokinetic variability [36] and potential relationship between serum drug concentration and therapeutic efficacy [23, 25] or toxicity [37]. On the other hand, the same guidelines do not support systematic TDM of fluconazole and echinocandins [33] because of the linear and predictable pharmacokinetic profile [31, 38] as demonstrated by studies performed in vitro and in healthy volunteers.

Nevertheless, when the pharmacokinetics of fluconazole, anidulafungin and caspofungin were prospectively addressed in critically ill patients receiving fixed doses of antifungals, considerable interindividual variability was observed, with a large proportion of patients (up to 33%) not attaining the optimal pharmacokinetic/pharmacodynamic target [13].

In the same sense, our data also demonstrate a high variability of antifungal exposure. Accordingly, it is not possible to predict a priori the antifungal concentrations achieved in a particular patient, suggesting that it may be necessary to ascertain the drug concentrations reached. Regarding this aspect, during the study period, 90.5% of patients receiving antifungals at our center were treated appropriately, according to current guidelines. However, only 67.1% had an adequate antifungal serum level, thus supporting the role of systematic TDM for optimizing antifungal treatment.

The high variability of the levels and low correlation between the dosage administered and serum concentration may be attributed to different aspects including inconsistent absorption [39, 40], body weight [41] and composition [42], genetic polymorphism and metabolism [43] and elimination [32] or interaction between different drugs [40] [44]. This may be especially relevant for specific types of patients, such as critically ill or hematologic patients [12, 13, 15], which represent the most important population in our cohort. An in-depth analysis of factors affecting the trough serum concentration of antifungals in our population showed that most of the inter-patient variability could be explained by demographic characteristics (age and body surface area). Although we found a significant positive correlation of antifungal dosage in the entire population, this fact was due to the patients receiving micafungin who were the only group with different doses. Our analysis also showed a positive correlation between serum albumin concentrations and fluconazole and echinocandin exposures. Nguyen et al. [16–26] reported that low serum albumin concentrations in the surgical ICU patients were correlated with low caspofungin exposures. We believe that patients with hypoalbuminemia should be considered a “high-risk group” of low antifungal serum exposure who could especially benefit from systematic monitorization of the antifungal concentration.

Many authors in the last 2 decades have tried to assess a relationship between serum antifungal exposure and clinical outcome [15–26, 45–49]. Although the benefit of voriconazole TDM was established by different studies [16–26], including a randomized controlled clinical trial [45], very few studies have analyzed the impact of fluconazole or posaconazole [14, 15, 50] TDM on the clinical outcome of patients with IFI. Moreover, to the best of our knowledge, studies evaluating this aspect in patients treated with echinocandins have not been performed yet. As for fluconazole, a retrospective study in The Netherlands including 99 critically ill children (46 with a proven invasive fungal infection) found a positive association between fluconazole trough concentration and a shorter time of culture conversion. In another study, Manosuthi et al. [14] examined 64 HIV-infected patients with cryptococcal meningitis treated with a combination therapy of fluconazole at different dosages plus amphotericin B. They found that patients with a high serum and CFS fluconazole concentration exhibited a higher rate of survival. Similarly, in a study performed in 17 patients receiving posaconazole (6 with probable/proven IFI), the authors found that a serum concentration ≥ 0.5 μg/ml was associated with a successful outcome [50].

Although not directly evaluated in this study, our findings suggest that an adequate serum antifungal concentration may be an additional tool for improving the clinical outcome of patients with suspected or confirmed IFI. Interestingly, this relationship seemed to be stronger when only patients receiving empirical or targeted antifungal therapy were analyzed. A possible explanation could be the fact that the wide use of active antifungal prophylaxis has significantly decreased to < 5% the rate of breakthrough IFI [51], which was the only factor associated with poor outcome in the prophylaxis subgroup. The inclusion of patients receiving prophylaxis might also explain why the proportion of patients with a favorable outcome in our cohort appears significantly higher compared with previous studies only focusing on patients with proven IFI [6].

The study has some limitations that should be addressed. First, our study conclusions are limited by the relatively small number of patients. Second, in this proof-of-concept study we evaluated the determination of only one antifungal concentration per patient, without estimating pharmacodynamic parameters. Third, although we used previously proposed cutoffs for fluconazole and echinocandin TDM [15, 31], we are aware that adequate trough serum concentrations for such drugs have not yet been established. Fourth, we did not record relevant drug-drug interactions that could explain, at least in part, the high intervariability observed. However, this factor could have been minimized by the existence of an alert system from the pharmacy department that makes an immediate notification about every possible drug-drug interaction. Lastly, the universal applicability of systematic TDM may be limited by the availability of laboratories. Strengths of our study include the fact that it was a prospective study performed in large hospitals and that it represents important real-life experience with TDM in hospitalized patients receiving antifungals.

## Conclusions

In summary, we show that with the standard antifungal dosage, a considerable proportion of patients have low drug concentrations, which are associated with poor clinical outcome. If future studies confirm these data, antifungal drug monitoring should be performed routinely in hospitalized patients and doses should be scheduled according to the levels reached.
